# Self-folding Structural Design Using Multiscale Analysis on the Light-absorption Folding Behaviour of Polystyrene Sheet

**DOI:** 10.1038/s41598-017-14599-z

**Published:** 2017-10-27

**Authors:** Yonghee Lee, Junghwan Moon, Joonmyung Choi, Maenghyo Cho

**Affiliations:** Division of Multiscale Mechanical Design, School of Mechanical and Aerospace Engineering, Seoul National University, San 56-1, Shillim-Dong, Kwanak-Ku, Seoul, 151-744 South Korea

## Abstract

Concentrated light-absorption on specific areas of polystyrene (PS) sheet induces self-folding behaviour. Such localized light-absorption control is easily realized by black-coloured line pattern printing. As the temperature in the line patterns of PS sheet increases differently due to the transparencies in each line pattern, localized thermal contraction generates folding deformation of the PS sheet. The light-activated folding technique is caused by the shape memory effect of PS sheet. The shape memory creation procedure (SMCP) is described by using molecular dynamic (MD) simulation, and the constitutive model of PS sheet is identified. This study employs the shell/cohesive line element for the folding deformation of PS sheet, and utilizes the constitutive model obtained from the MD simulation. Based on the continuum-model analysis of the PS sheet folding deformation activated by light, various self-folding structures are designed and manufactured.

## Introduction

Self-folding technique, which does not require additional activation devices, is achieved by using smart materials, and it is applied to various industrial fields, such as robotic systems^1–11^, MEMS^12–14^, biomedical devices^15^, solar power cells^16^, sensors^17,18^, and drug delivery systems^19–23^. The commonly used smart materials for self-folding are shape-memory polymers (SMPs), electric devices with metalized polyester film (MPF)^24^, and patterned composites using stiffness differences^25^. Such self-folding techniques show good performance and stale behaviour, but they require direct energy supply for activation. Recently, a light-activated self-folding technique using transparent polystyrene (PS) sheet was developed that received much attention^26–30^. The PS sheet is easily folded by light-absorption, so various 3D shapes can be designed by self-folding design of the PS sheet. The PS sheet is manufactured under high temperature and pressure, so it shrinks to (50–60) % when it approaches the glass transition state^31–37^. As the heat is absorbed in the specific narrow area of the PS sheet, the sheet is folded by the localized heat-contraction. Such localized thermal absorption is easily accomplished by the light and black-line pattern printing. When light shines on the transparent PS sheet, most of the light would pass through the sheet. However, the black-coloured area absorbs light, including infrared ray; and then the folding deformation occurs, due to the glass transition state in the specific area. The self-folding technique using PS sheet uses the light as activation energy, so that non-contact control is possible. Also, there are more advantages in using the light-activated self-folding behaviour of the PS, such as non-harmful energy source, low cost, and low activation energy.

In continuum scale analysis, the curved deformed geometry of the self-folding PS sheet should be expressed. Accordingly, the basic mechanical behaviour of the polystyrene sheet can be analysed using the shell element formulation with curvilinear coordinate system. In addition, the folding deformation at the black-coloured line pattern is caused from extreme deformation in the narrow area. This means that accurate description of the folding deformation of the PS sheet requires discontinuous folded angles at both sides of the folded line, which is different with bending behaviour. Therefore, a cohesive line element is implemented for predicting the displacement and folding angle discontinuity. The cohesive line element provides the additional nodes at the folded line to generate the discontinued rotation angles at localized heated region. Giampieri and Perego^38,39^ used the cohesive line element for the analysis of the paperboard folding behaviour, and showed that it can solve the difficulty of the analysis caused from the discontinued rotation angle at the folded line. Applying the cohesive line element to the shell element^40,41^, the folding deformation of the PS sheet can be predicted.

The precise prediction and design of the self-folding deformation can be achieved by a multiscale integrated approach, which reflects the microscopic origin of the phenomenon. The polystyrene is a kind of shape memory polymer, and it requires specific manufacturing procedure to realize the shape memory effect (SME). This cyclic thermomechanical treatment, which is called the shape memory creation procedure (SMCP), consists of sequential steps. First, an external load is applied at above the glass transition temperature (${T}_{g}$). The macroscopic stretching induces the microscopic alignment of the polymer chain and corresponding anisotropy of mechanical stiffness. Then, the desired shape is fixed by cooling and stress relaxation steps. Finally, pre-stretched shape recovers to the original shape by heating, and it is induced by entropic elasticity of the polymer chains. Notably, the extent of shape recovery is significantly affected by the thermomechanical history during the pre-stretching stage. The anisotropy of mechanical properties also can be varied with respect to the temperature and pre-applied deformations. However, it is not easy to evaluate the mechanical response of the shape memory polystyrene with consideration of these internal and external parameters.

Molecular dynamics (MD) simulation can be a powerful tool to provide a relation between the microscopic state and thermoelastic behaviour. Some researchers have performed the MD simulation and micromechanics-based theoretical analysis of the PS sheet considering the shape memory effect^42,43^. In particular, our previous research^42^ described the thermomechanical cycle of polystyrene, and quantified the shape memory properties in molecular scale via MD simulation. From a microscopic-structural viewpoint, it concentrated on observing temporal changes of the shape with respect to initially defined molecular structure.

This paper expands the previous work to provide multiscale analysis methodology and design parameters for self-folding deformation of the PS. The effect of polymer chain orientation on the folding angle is reflected by establishing a relationship between pre-applied strain and in-plane thermal contraction. Moreover, in order to monitor temporal evolution of mechanical response and construct the mechanistic constitutive model, the stress-strain relationship is parameterized with temperature and programmed strain. Continuum scale analysis employs the shell/cohesive line element and the information obtained from the MD simulation to precisely identify the self-folding deformation of polystyrene sheet.

## Multiscale continuum model analysis of self-folding structures

**Figure 1:**
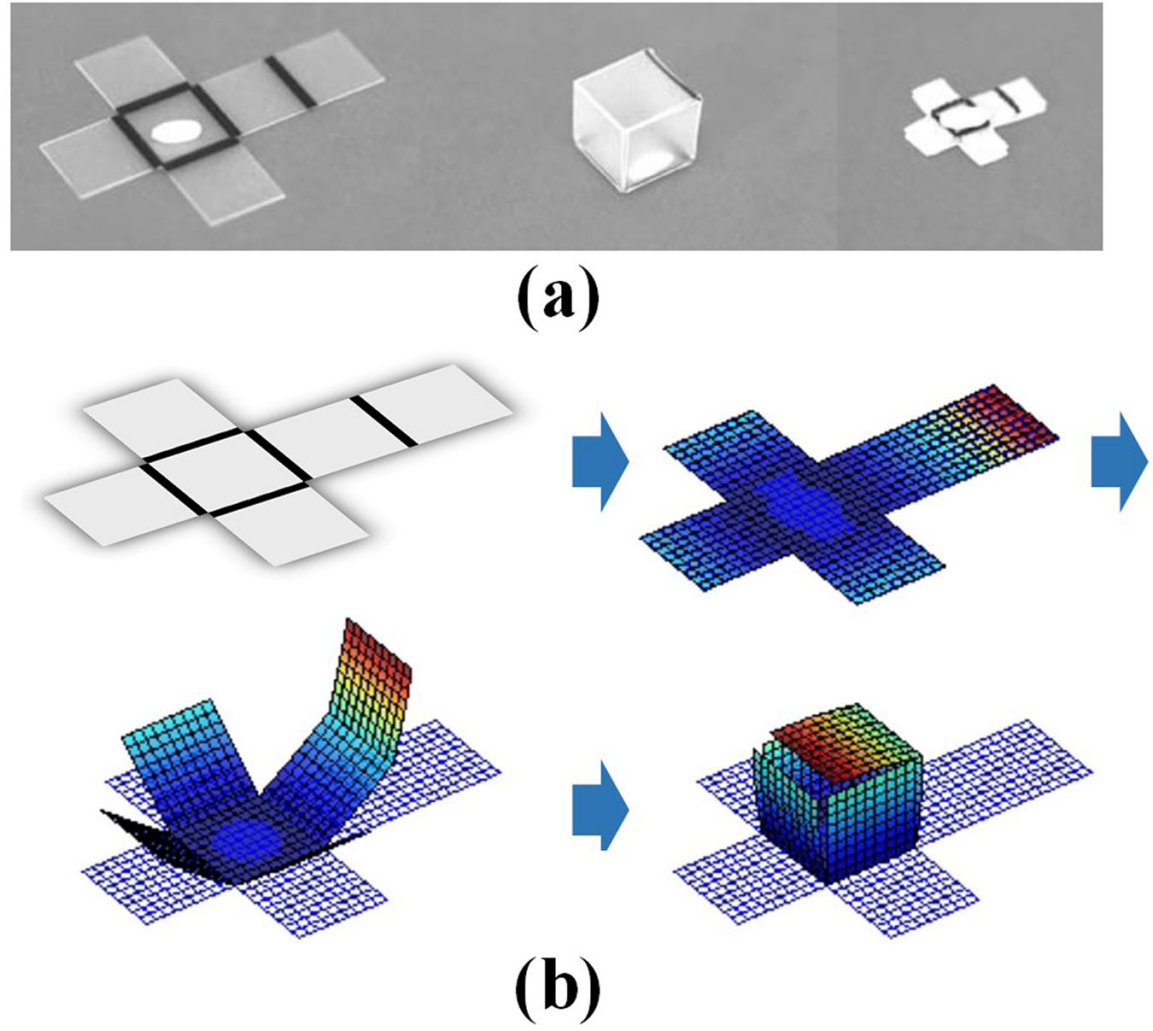
Self-folding structure with hexahedral shape (**a**) Actual model, and (**b**) Folding simulation using multiscale continuum-model.

The self-folding structure using the PS sheet is activated by light including infrared ray, and its folding behaviour can be predicted from the continuum model analysis mentioned in the next chapter. The folding behaviours of some self-folding structure models are estimated by the continuum model analysis. The constitutive deformation models of polystyrene obtained from MD simulations are applied to the continuum model analysis. Figure 1 shows the simple hexahedral shape using PS sheet that is first performed. The self-folding structure with hexahedral shape can be easily designed as a self-folding structure, and the fabrication of the actual model is performed in Liu *et al*.’s research^26^. The folding angle of the hexahedron is 90°, so the line pattern width is determined to be 2.1 mm. The rectangular cell is set to the cell size 2 × 2 cm, and the PS sheet has the thickness 0.23 mm. Because each line pattern simultaneously starts folding deformation, the transparencies of the line patterns are the same as 0%. The surrounding temperature is fixed as 40 °C, and the folding deformation starts at the glass transition temperature of polystyrene sheet of 102 °C.

**Figure 2:**
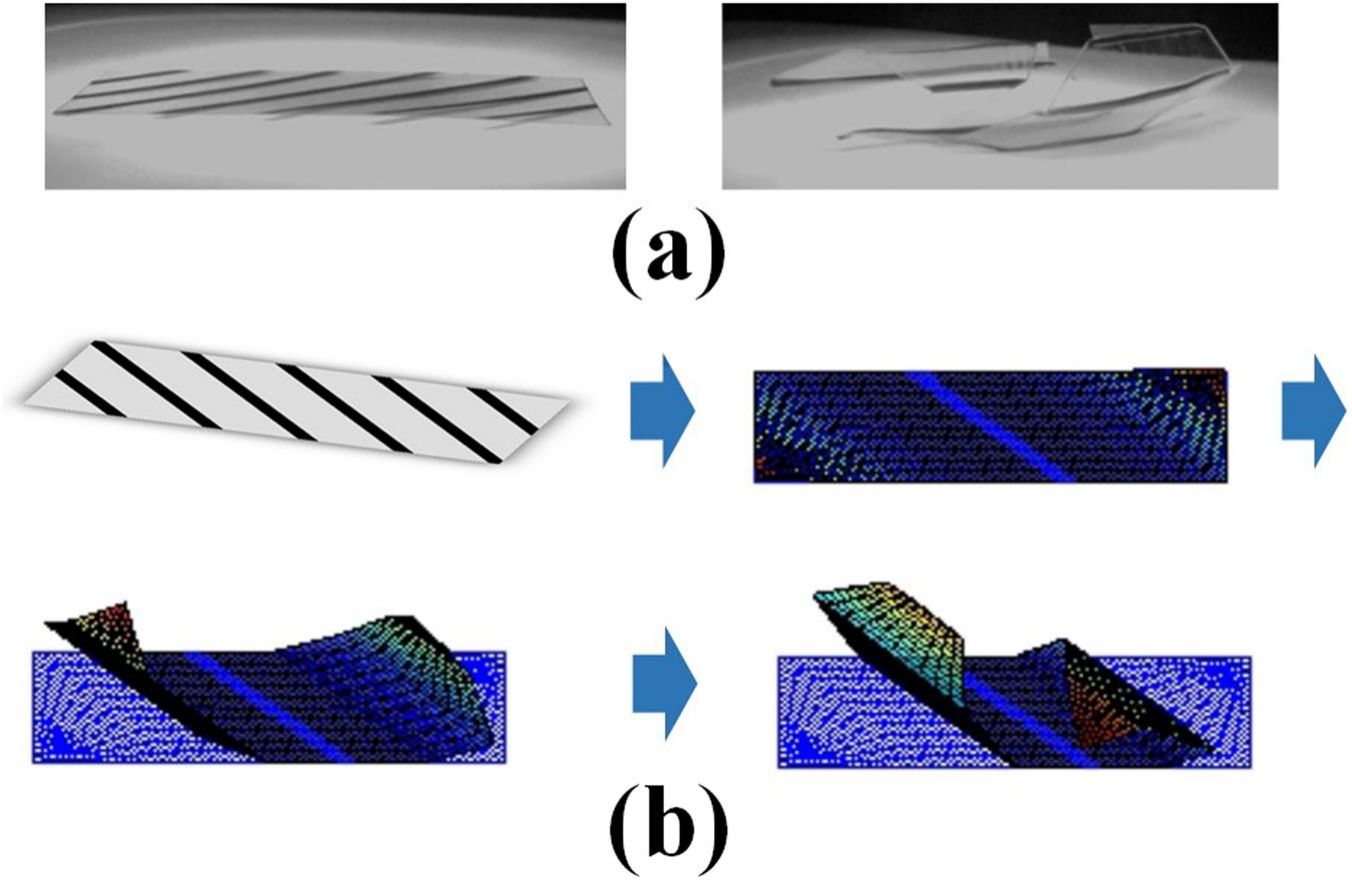
Self-folding structure with twisted shape (**a**) Actual model, and (**b**) Folding simulation using multiscale continuum-model.

Figure 2 shows the actual model and deformation process of the second model, which is a self-folding structure with twisted shape. The line patterns are arranged in parallel, and the PS sheet with rectangle shape is cut in the diagonal direction. As for the hexahedral shape model, the line pattern width is set to 2.1 mm, due to its folding angle of 90°, and the model is designed to the size 2 × 8 cm.

**Figure 3:**
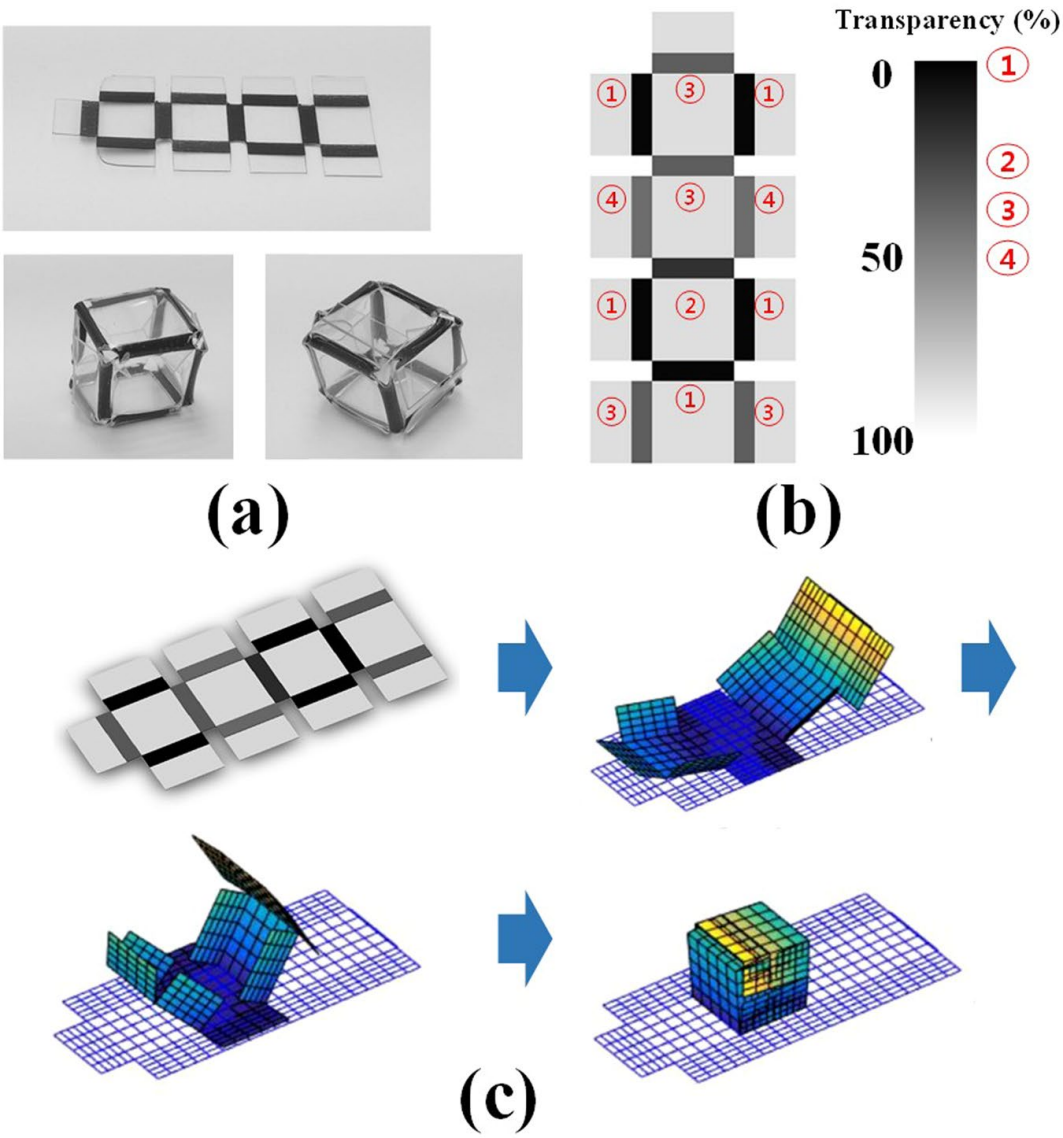
Hexahedral shape using sequential folding (**a**) Actual model, (**b**) Transparency at line patterns, (**c**) and Folding simulation using the multiscale continuum-model.

The last model is of hexahedral shape using sequential folding behaviour. As aforementioned, the self-folding structure with complex shape requires precise folding sequences between the respective line patterns. Accordingly, although the self-folding structure with hexahedral shape such as Fig. 1 shows successful simulation result, the actual model can sometimes fail to reach the planned shape. For stable self-folding success of the structure with complex shape, a sequential folding technique such as Fig. 3 is considered^30^. The final shape in Fig. 3(c) is the same as the model in Fig. 1(a), but its stability is higher than the simple hexahedral shape, due to the collar faces attached to the edge line of the PS sheet, and the sequential folding design. Because the faces of the PS sheet have to be folded at their folding sequences, the respective transparencies are differently applied to each of the line patterns, as in Fig. 3(b). In the line patterns, the time to approach glass transition temperature is determined by the transparency, so the faces can be sequentially folded.

## Conclusion

The self-folding behaviour of a PS sheet activated by light irradiation is designed by fabrication of the line pattern and corresponding localized light absorption. Especially, in order to maximize the applicability of non-contact actuation, sequential folding structure is manufactured by modifying transparency of the pattern. Because the self-folding functionality is induced by shape recovery of microscopic polymer chain, MD-based multiscale approach is developed for precise prediction and design. The shape-memory cycle for polystyrene sheet is performed, and then the behaviour of the pre-strained polystyrene sheet is estimated at the nanoscale. The influence of pre-strain on thermal in-plane shrinkage is identified, and it is implemented to the continuum model. Also, elastic and hardening moduli along the pre-stretched direction are estimated as a function of internal and external parameters. These are used to provide the simplified linear elasto-plastic deformation model. In continuum scale folding model, the cohesive line element is employed to describe the discontinued rotation angle at the folded line. The final deformed configuration obtained via multiscale framework shows a good agreement with that of actual experiment. We expect our results can be used to design various complex shape and precisely control sequential deformation of the self-folding structure.

## Methods

**Figure 4:**
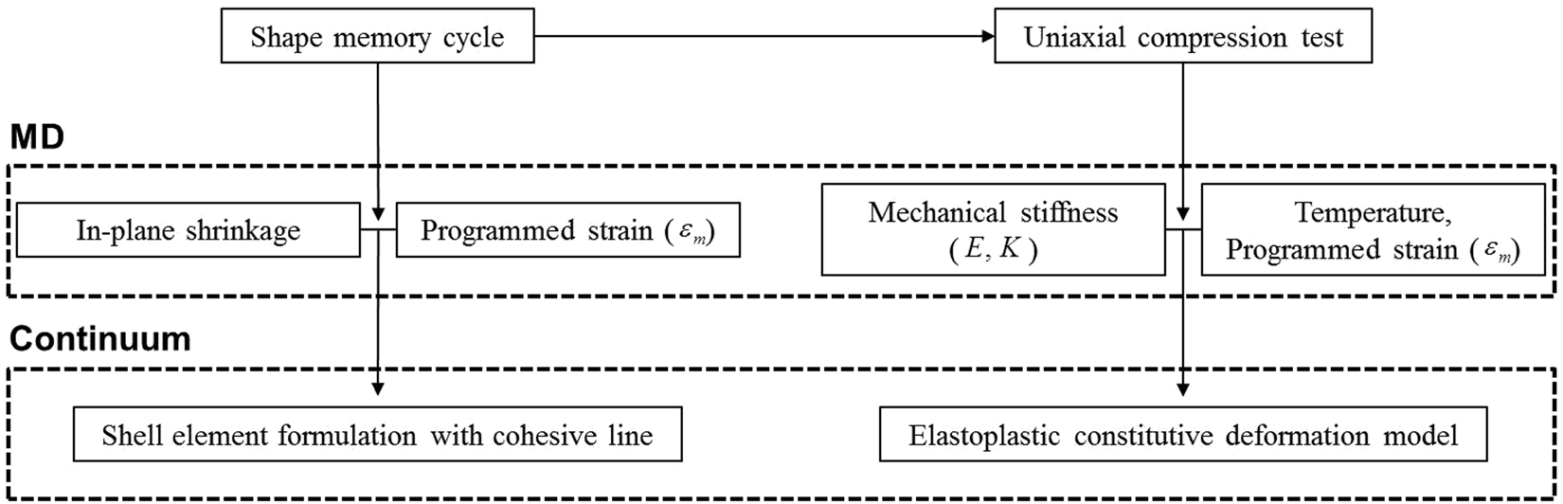
Scheme of the multiscale analysis of mechanical response of the self-folding PS.

Figure 4 shows a schematic diagram of the multiscale framework which connects the molecular shape recovery motion and continuum scale folding deformation of the PS. First, the shape memory cycle is described by the MD simulation. The dependence of thermal shrinkage on initially applied strain is quantified by monitoring change in the shape of unit cell during thermal transition. The microscopic information is transferred to continuum scale analysis which implements the finite shell and cohesive line elements to predict the folding angle. Then, uniaxial compressive loading is applied to each deformed MD unit cell to get the stress-strain curve. We propose simple equations to estimate the elastic and hardening moduli as a function of the temperature and initially defined shape. These are utilized to construct the simplified bilinear elasto-plastic constitutive deformation model. This section introduces the computational methodology of both molecular dynamics and continuum simulation to provide the prediction model of mechanical response of the self-folding PS.

### Multiscale constitutive model construction using molecular dynamic simulation

This section describes the atomistic modelling and MD simulation methodology to describe the shape memory creation cycle to impose the shape memory effect to the PS polymer chains, and implement the microscopic design parameters into the continuum-scale model. Our previous study^42^ revealed that only pre-stretched PS shows self-folding behaviour at the glass transition temperature. This is because the main mechanism of the thermal contraction is the shape recovery from anisotropic to isotropic state. The previous results also showed that the thermoelastic properties and final recovered configuration of the self-folding PS are determined by the shape-programming path. In this study, the linear elasto-plastic deformation model of the oriented PS sheet is expressed as a function of two key parameters, which are the pre-imposed strain, and the temperature. Furthermore, the relation between the pre-strain applied to the PS, and its local shrinkage, is quantified, using the MD calculations and experimental values. This relation is implemented to the continuum scale prediction of the macroscopic folding angle and final deformed configuration.

Atomistic modelling of the initial bulk PS unit cell and relaxation simulation are performed by using Materials Studio 5.5 (Accelys, Inc.), which uses the Teodorou-Suter method to construct the amorphous structure^44^. The intramolecular and intermolecular interactions are described by the polymer consistent force field (PCFF)^45^. This can identify the thermomechanical properties and glass transition behaviour of the polymeric materials, because the potential coefficients are derived from the experimental properties of the organic compounds. The atom-based summation with a cutoff distance of 9.5 Å is used to calculate the non-bonded interaction between atoms. In treating the Coulombic interaction, the distance-dependent dielectric method with a dielectric value of 2.6^46^ is used.

**Figure 5:**
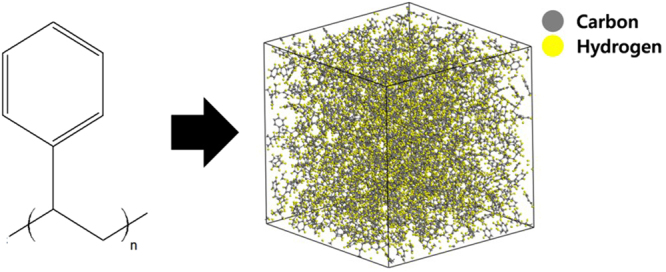
Configuration of the relaxed bulk PS unit cell that is modelled using MD simulation.

The MD unit cell consists of atactic PS polymer chains, and the periodic boundary condition is applied to every surface of the unit cell for modelling the bulk system. In order to ensure the physical crosslinking between chains, the molecular weight of the PS should be larger than its entanglement molecular weight (${M}_{e}$ = 19,000 g/mol). The entanglement is required to realize the shape recovery deformation, because it plays the role of a net point for fixing the original shape. Therefore, two polymer chains with a molecular weight of 20,831 g/mol each (400 repeating styrene units) are used to construct the unit cell. Energetically favourable structure is made by applying a sequential equilibration procedure: energy minimization using the conjugated-gradient method, NVT ensemble for 500 ps at 300 K, and the NPT ensemble for 2.5 ns at 300 K and 0.1 MPa. The time step increment is 1.0 fs. In order to ensure that the polymer chains are distributed without any anisotropy, the annealing simulation, including five cycles of the NVT ensemble for 250 ps, is performed. The temperature reduces from 900 to 725 K at each cycle, and the scalar orientation order parameter of the PS backbone chains (this will be explained below, see also Eq. 1) converges to zero after the annealing process. Figure 5 shows the configuration of the amorphous bulk unit cell.

Prior to applying the shape memory creation treatment, our model is utilized to calculate the thermoelastic properties to confirm sufficient equilibration and validate the reliability of simulation methods. The glass transition temperature and linear coefficient of thermal expansion (CTE) of relaxed unit cell are determined by monitoring the variations of specific volume and mean square displacement (MSD) of the system within the temperature range of 300–500 K. The relaxed structure at 500 K and 0.1 MPa is cooled to 300 K with a cooling rate of 10 K/ns under isobaric condition. Then, the specific volume-temperature plot is obtained by averaging the specific volume for the last 100 ps of the NPT equilibration steps (1 ns) at each temperature. The candidate of ${T}_{g}$ within the simulated temperature range is approximated as the temperature at which the MSD of the system abruptly increases. It is because the glassy-rubbery transition induces huge increase in the chain mobility. The approximated point is used to divide the glassy and rubbery states of the unit cell, and ${T}_{g}$ is defined as the intersection of the linear regression lines at each region. The linear CTE can be obtained by using the slope of specific volume-temperature plot. The procedure to characterize the glass transition and calculate the thermoelastic properties is described in our previous study in detail^42^. These properties showed good agreement with experimental values.

**Figure 6:**
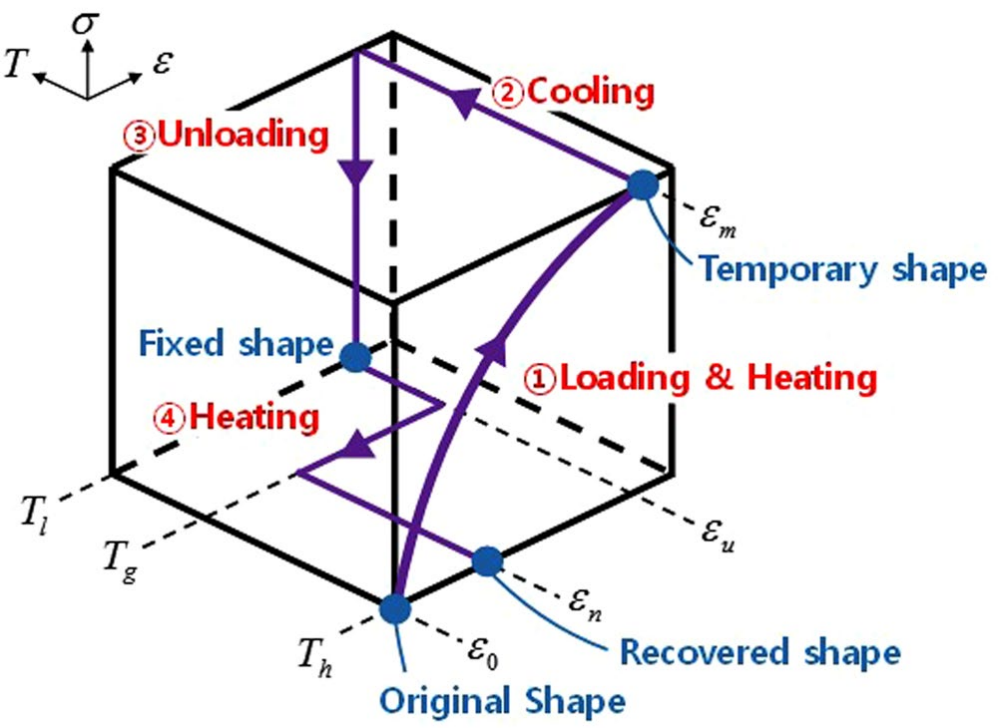
Scheme of the shape memory creation procedure applied to the PS conducted in MD simulation.

The thermomechanical cycle is applied to the initial isotropic polymer chains, in order to produce the shape memory effect. All MD simulations describing the shape memory cycle are carried out using the LAMMPS code developed by Sandia National University^47^. The relaxed unit cell is heated up to ${T}_{h}$ = 550 K, above the thermal transition temperature (${T}_{g}$ = 383 K and ${T}_{m}$ = 513 K), by applying an equilibration step, which consists of energy minimization, NVT, and NPT ensemble simulations for 1.0 ns, respectively. After the heating up process, the thermomechanical test begins by biaxial stretching the PS chains along the in-plane directions (*x* and $y$) with a nominal strain rate of ${10}^{8}/{\rm{s}}$. The deformed states with six different programmed strains (${\varepsilon }_{m}$ = (15, 30, 45, 60, 80, and 100) %) are prepared to estimate the relation between the initial molecular anisotropy and corresponding mechanical deformation behaviour, and the extent of thermal shrinkage. Then, each MD cell is cooled down to ${T}_{l}$ = 300 K using the NVT ensemble for 1 ns, and the external loading is removed by using the NPT ensemble for 1 ns, respectively. During the stress relaxation, the nominal strain slightly decreases to the fixed strain (${\varepsilon }_{u}$). Final in-plane thermal shrinkage of the oriented PS is realized by re-heating it with a rate of 5 K/ns under isobaric condition. The unit cell is heated up to the temperature at which the molecular chains recover to the isotropic state ((550–650) K). During contraction to the recovered shape (${\varepsilon }_{n}$), the cell lengths along all three dimensions are controlled independently to reflect the anisotropic movement of the chains. The time step for the entire thermomechanical cycle is 0.5 fs. Figure 6 shows the entire scheme of the thermomechanical cycle conducted in the presented MD simulation study.

Entropic shape recovery is caused by microscopic conformational transformation of the polymer chains. This microscopic motion and corresponding anisotropic/isotropic transition during shape memory cycle can be effectively captured using orientational order parameter (${P}_{i}$)^42^, which can be calculated via:

1 $${P}_{i}=\frac{3}{2}\langle {\cos }^{2}{\theta }_{i}\rangle -\frac{1}{2}.$$

**Figure 7:**
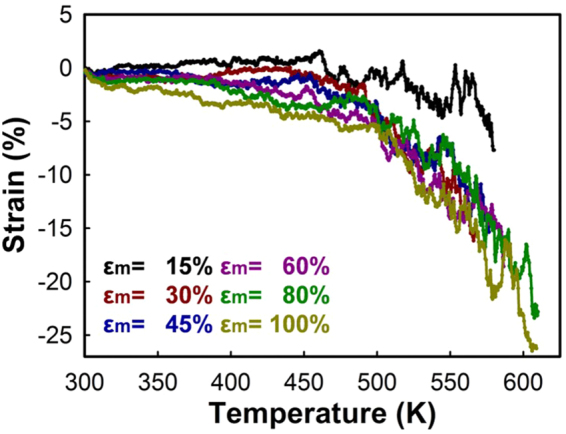
Thermal contraction strain of the oriented PS with different programmed strains.

Here, ${\theta }_{i}$ is the angle between the *i*–axis of the Cartesian coordinate, and the vector of the polymer backbone segments. The polymer chains are perfectly isotropic when ${P}_{i}$ equals to zero, and they become in aligned state as ${P}_{i}$ approaches 1.0. As the PS unit cell is stretched up to ${\varepsilon }_{m}$ = 100%, the order parameter along the loading direction increases gradually up to 0.1. All the unit cells with different ${\varepsilon }_{m}$ are heated and Fig. 7 shows corresponding in-plane thermal contraction strain of the oriented PS with respect to temperature. The lengths of unit cells are monitored, until the polymer structure is fully recovered to the isotropic state (${P}_{i}$ = 0). As the pre-applied strain increases, the shape recovery ratio (${R}_{r}=({\varepsilon }_{m}-{\varepsilon }_{n})/{\varepsilon }_{m}$) decreases. This is because the unrecoverable strain and disintegration of the polymer network occur with larger deformation. However, Fig. 7 shows that the total shrinkage increases as the unit cell is more deformed. Table 1 shows microscopic order parameter and shape memory properties of the oriented PS with different pre-strain. Previously, we have established a relation between the initial programmed strain and total in-plane shrinkage by using MD data points, and this can predict the experimental results with larger ${\varepsilon }_{m}$ ^48^. This relation is used to predict final folding angle of the localized heated region of the oriented PS sheet.

**Table 1:** Orientational order parameter and shape memory properties of the oriented PS with different initial strains.

Programmed strain (%)	Orientational order parameter	Shape recovery ratio (%)	In-plane thermal strain (%)
15	0.021	80.336	8.315
30	0.049	72.408	14.353
45	0.064	56.396	15.263
60	0.071	54.165	20.153
80	0.085	48.707	21.635
100	0.105	48.893	26.570

The results were obtained via molecular dynamics simulation^42^.

The operating temperature for shape recovery deformation estimated in the MD simulation is slightly higher than the experimental ${T}_{g}$ ((382–388) K). The temperature shifting is due to the limitation of short time-scale, which cannot observe all of the conformational recoveries at the experimentally suggested temperature. However, our simulation schemes can clearly observe the shape changes induced by thermal transition of the polymer chain molecules. Furthermore, in contrast to the instantaneous heating technique^49,50^, the long-time gradual heating used in this paper has the advantage of characterizing the dependence of the microstructural parameters on the macroscopic self-folding behaviour of the PS.

The macroscopic self-folding angle and final deformed configuration of the PS sheet are considerably varied by the local temperature distribution and intrinsic mechanical anisotropy of the irradiated region. Therefore, the compressive mechanical response of the pre-stretched PS is parametrically studied in terms of the operating temperature and pre-strain using the MD simulation. Each pre-deformed MD unit cell (${\varepsilon }_{m}$ = (0, 30, 60, 80) %) after stress relaxation at 300 K is compressed along the in-plane directions with a constant strain rate of 10^8^ /s under the NPT ensemble. The applied strain ranges up to 15%, and it is known that both elastic and plastic deformations of the PS can be clearly seen within this strain range^51^. The virial formula is used to calculate the stress at each deformed state, and the operating temperature ranges from (300 to 440) K. The results from three MD runs with different initial atomic velocity distribution are averaged to present the stress-strain relationship of the PS.

**Figure 8:**
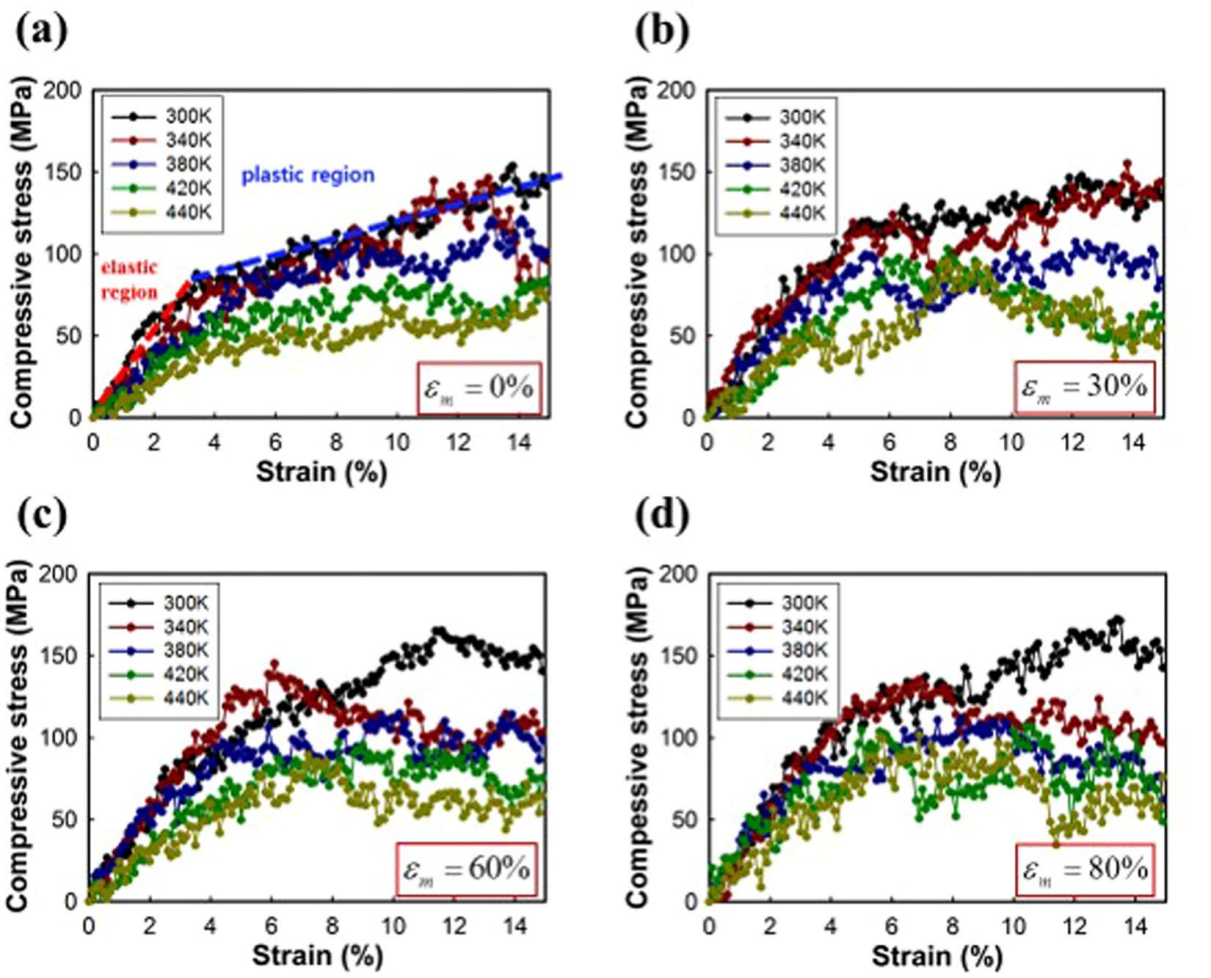
Compressive stress-strain curves of the polystyrene as a function of temperature.

Figure 8 shows the change of stress-strain curve with temperature and programmed strain. Figure 8(a) shows that the mechanical response of the PS to the uniaxial compressive loading can be assumed as linear elasto-plastic behaviour. The linear elastic regime lasts within a strain range of $\varepsilon$ = (0–3) %, and yielding occurs at $\varepsilon$ = 4%. To simplify the model, the dependence of the yield point on the temperature and the morphology of the oriented PS are not considered. As the temperature rises, the slope of curve at the elastic regime (elastic modulus ($E$)) is almost linearly decreased. In the case of the non-oriented system, $E$ decreases with a rate of 10.3 MPa/K at low temperature below ${T}_{g}$. This decreasing rate is reasonable, compared with other literature^51^, which is of the same order as the experimental value (5 MPa/K). With heating up above the glass transition temperature, the modulus drops to about 1.38 GPa at the viscous rubbery regime. The decreasing tendency of the modulus in this measurement can also be clearly seen in the plastic deformation. The slope of the s–s curve at the plastic regime ($K$) is about 0.6 GPa at 300 K, which is 77% smaller than the elastic modulus. As the temperature rises within the (300–380) K range, $K$ decreases with a rate of 3.9 MPa/K, and drops to 0.25 GPa at the rubbery state.

**Figure 9:**
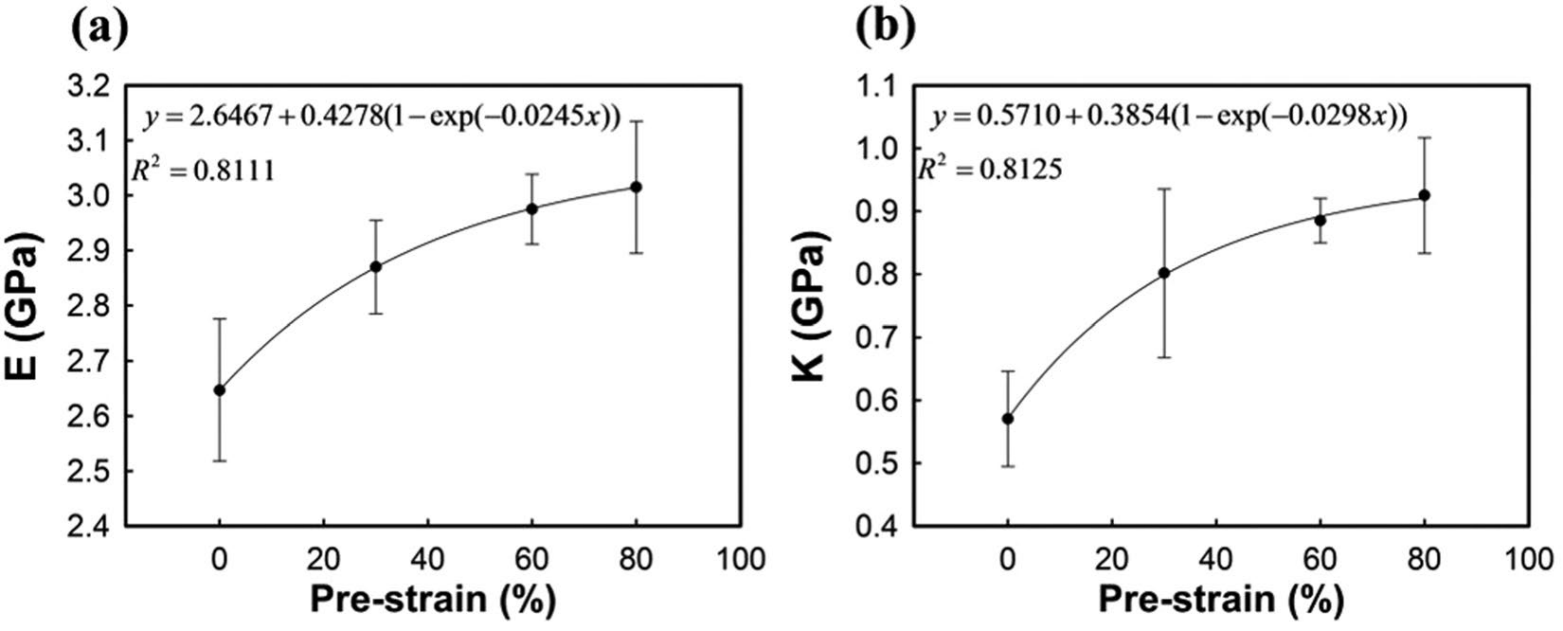
The slopes of compressive stress-strain curves at the (**a**) elastic, and (**b**) plastic region at $T$ = 300 K. The solid line represents the fitted function with respect to the pre-strain.

The influence of mechanical anisotropy on the deformation behaviour, dominantly determined by the extent of the pre-applied strain, is also quantified. Figure 9(a) and (b) show variations of tangential stiffness at the elastic and plastic regions at 300 K, respectively. The mechanical properties ($E$ and $K$) are increased with increasing the pre-strain. The biaxial in-plane stretching applied during the manufacturing process of the oriented PS imposes molecular orientation on the macromolecular chains. Therefore, the increase of mechanical anisotropy with greater extent of pre-stretching is evident, even within the fully plastic regime, as well as the linear elastic regime. $E$ and $K$ are fitted by an exponential rise to the optimal model, because they converge to specific value. The solid lines in Fig. 9(a) and (b) are the fitted curves, and the square of the correlation coefficients is about 0.81.

In order to construct the constitutive model with general expression, two slopes of the s–s curve at different regimes are expressed as a function of the temperature and pre-strain. In order to make a simple model, the influences of these two input variables are independently considered, and hence it is assumed that the decreasing rates of modulus with respect to the temperature are all the same, regardless of the pre-strain. The final prediction equation for $E$ and $K$ at temperature below ${T}_{g}$ can be expressed as:

2 $$E({\varepsilon }_{m},T)=5.7404+0.4278(1-\exp (-0.0245{\varepsilon }_{m}))-3.9187(T/{T}_{g})\,({\rm{Gpa}}).$$

3 $$K({\varepsilon }_{m},T)=1.7553+0.3854(1-\exp (-0.0249{\varepsilon }_{m}))-1.5001(T/{T}_{g})\,({\rm{GPa}}).$$

**Figure 10:**
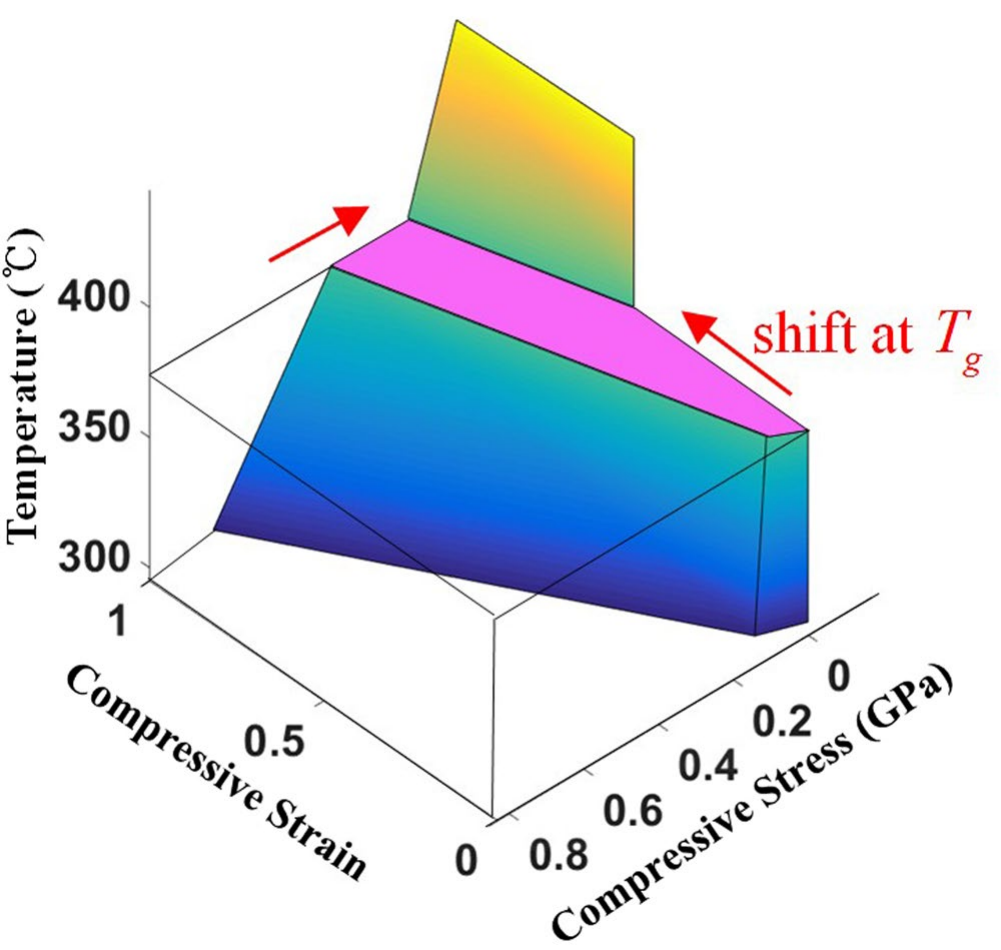
Stress-Strain-Temperature curve of polystyrene sheet used in this study with ${\varepsilon }_{m}$ = 200%.

For precise prediction of the self-folding deformation, it is important to predict how much in-plane contraction will occur at the stage of glassy-rubbery transition of the polymer. As discussed above, the extent of shape recovery has close relation to the degree of anisotropy of the microstructure. Our previous work^42^ provides a model to expect total in-plane shrinkage of the oriented PS as a function of the programmed strain with practical range (${\varepsilon }_{m}$ = (150–300) %). For example, the polystyrene sheet used in our study shows shrinkage of about 50% compared with the original shape, and its pre-strain can be estimated to be 200% using this model. Because the folding behaviour of the oriented PS occurs without any external mechanical loading, the stress-strain curve is shifted by the amount of the predicted contraction at $T={T}_{g}$. The deformation at temperature above ${T}_{g}$ is regarded as plastic deformation, because the self-folding of PS is not recoverable, unless another thermomechanical cycle is applied to it. Combining the MD simulation-based prediction of mechanical parameters and in-plane shrinkage together, the constitutive deformation model with various pre-strain and temperature is constructed just as shown in Fig. 10:

### Shell finite element

This section employs the shell element used in Brank *et al*.’s research^40,41^. The shell director is defined using an Euler rotation matrix, and it is assumed as the vector with magnitude 1, which is defined as a unit sphere. The shell director is expressed by the combination of two Euler angles, which are the rotation angles $\alpha$ and $\beta$. The rotation angles $\alpha$ and $\beta$ are defined from the *x*-axis and *y*-axis rotation, respectively. In the undeformed configuration, it is assumed that the *x-y* axis rotation angles are initially zero. Then, the relationship between the undeformed shell director $\overrightarrow{T}$ and the deformed shell director $\overrightarrow{t}$ follows as:

4 $$\overrightarrow{t}=Q(\alpha ,\beta )\overrightarrow{T}.$$

The rotation matrix *Q* is determined by the rotation angles $\alpha$ and $\beta$, and is separated into two parts, ${Q}_{1}(\alpha )$ and ${Q}_{2}(\beta )$. Each matrix has rotation angles on its respective rotation axis (*x,y*). Also, if the undeformed shell director $\overrightarrow{T}$ is assumed to 0 0 1, which is orthogonal to the surface, the deformed shell director $\overrightarrow{t}$ can be expressed as:

5 $$\begin{array}{rcl}\overrightarrow{t} & = & {Q}_{2}(\beta ){Q}_{1}(\alpha )\{\begin{array}{c}0\\ 0\\ 1\end{array}\}\\  & = & [\begin{array}{ccc}\cos (\beta ) & 0 & -\sin (\beta )\\ 0 & 1 & 0\\ \sin (\beta ) & 0 & \cos (\beta )\end{array}][\begin{array}{ccc}1 & 0 & 0\\ 0 & \cos (\alpha ) & -\sin (\alpha )\\ 0 & \sin (\alpha ) & \cos (\alpha )\end{array}]\{\begin{array}{c}0\\ 0\\ 1\end{array}\}\\  & = & \{-\cos (\alpha )\sin (\beta )-\sin (\alpha )\,\cos (\alpha )\cos (\beta )\}\end{array}.$$

First, the undeformed/deformed position vectors are expressed as:

6 $$\overrightarrow{R}({\theta }^{i})=\overrightarrow{X}({\theta }^{\alpha })+{\theta }^{3}\overrightarrow{T}({\theta }^{\alpha })\overrightarrow{r}({\theta }^{i})=\overrightarrow{x}({\theta }^{\alpha })+{\theta }^{3}\overrightarrow{t}({\theta }^{\alpha }),$$

where, $\overrightarrow{R},\overrightarrow{X}\,\,{\rm{and}}\,\,\overrightarrow{T}$ mean the position vector, mid-surface position vector, and shell director defined in the undeformed configuration, respectively; $\overrightarrow{r},\overrightarrow{x}\,\,{\rm{and}}\,\,\overrightarrow{t}$ denote the position, mid-surface position vector, and shell director in the deformed configuration, respectively; and $\theta$ is the curvilinear coordinates. Greek indices are 1 and 2, while Latin indices run from 1 to 3. The displacement vector defined from the mid-surface position vectors has the following relationship:

7 $$\overrightarrow{x}({\theta }^{\alpha })=\overrightarrow{X}({\theta }^{\alpha })+\overrightarrow{u}({\theta }^{\alpha }).$$

The displacement vector $\overrightarrow{w}$ is induced from the position vectors as:

8 $$\overrightarrow{w}({\theta }^{i})=\overrightarrow{r}({\theta }^{i})-\overrightarrow{R}({\theta }^{i})=\overrightarrow{u}({\theta }^{\alpha })+{\theta }^{3}(\overrightarrow{t}({\theta }^{\alpha })-\overrightarrow{T}({\theta }^{\alpha })),$$

where, the vector $\overrightarrow{\square }$ is expressed as $\overrightarrow{\square }={\square }^{i}{\overrightarrow{g}}_{i}$ in the curvilinear coordinate system, and ${\square }^{i}$ is the contravariant component.

The covariant basis vectors are defined at the material point as:

9 $${\overrightarrow{G}}_{i}=\frac{\partial \overrightarrow{R}}{\partial {\theta }^{i}},{\overrightarrow{g}}_{\iota }=\frac{\partial \overrightarrow{r}}{\partial {\theta }^{i}}{\overrightarrow{G}}_{\alpha }={\overrightarrow{X}}_{,\alpha }+{\theta }^{3}{\overrightarrow{T}}_{,\alpha }\quad {\overrightarrow{G}}_{3}=\overrightarrow{T}{\overrightarrow{g}}_{\alpha }={\overrightarrow{x}}_{,\alpha }+{\theta }^{3}{\overrightarrow{t}}_{,\alpha }\quad {\overrightarrow{g}}_{3}=\overrightarrow{t},$$

where, ${\overrightarrow{\square }}_{i}$ means the covariant base vector. In addition, the metric tensors in the undeformed/deformed configuration are obtained. Using the metric tensor, the Green-Lagrangian strain tensor separated into the membrane, bending and transverse shear strains, is obtained as:

10 $$\begin{array}{rcl}{\varepsilon }_{\alpha \beta } & = & \frac{1}{2}({\overrightarrow{X}}_{,\alpha }\cdot {\overrightarrow{u}}_{,\beta }+{\overrightarrow{X}}_{,\beta }\cdot {\overrightarrow{u}}_{,\beta }+{\overrightarrow{u}}_{,\alpha }\cdot {\overrightarrow{u}}_{,\beta })\\ {\kappa }_{\alpha \beta } & = & \frac{1}{2}({\overrightarrow{X}}_{,\alpha }\cdot ({\overrightarrow{t}}_{,\beta }-{\overrightarrow{T}}_{,\beta })+{\overrightarrow{X}}_{,\beta }\cdot ({\overrightarrow{t}}_{,\alpha }-{\overrightarrow{T}}_{,\alpha })+{\overrightarrow{u}}_{,\alpha }\cdot {\overrightarrow{t}}_{,\beta }+{\overrightarrow{u}}_{,\beta }\cdot {\overrightarrow{t}}_{,\alpha }).\\ {\gamma }_{\alpha } & = & \frac{1}{2}({\overrightarrow{X}}_{,\alpha }\cdot (\overrightarrow{t}-\overrightarrow{T})+{\overrightarrow{u}}_{,\alpha }\cdot \overrightarrow{t})\end{array}$$

Total energy can be expressed by the Green-Lagrange strain and the second Piola-Kirchhoff stress tensor *S*. Then, by the approximated shell theory, the internal energy $U={\int }_{V}\frac{1}{2}{S}^{ij}{E}_{ij}\sqrt{g}dV$ becomes:

11 $$U={\int }_{A}\frac{1}{2}{n}^{\alpha \beta }{\varepsilon }_{\alpha \beta }+\frac{1}{2}{m}^{\alpha \beta }{\kappa }_{\alpha \beta }+{q}^{\alpha }{\gamma }_{\alpha }\sqrt{a}dA.$$

where, *A* is the mid-surface area. The covariant base vector ${\overrightarrow{a}}_{\alpha }$ is induced from ${\overrightarrow{a}}_{\alpha }=\frac{\partial \overrightarrow{x}}{\partial {\theta }^{\alpha }}$. If the high-order term of ${\theta }^{3}$ is neglected in the $\overrightarrow{g}$ and $\overrightarrow{a}$ formulation, the assumption $\sqrt{g/a}=1+$ $\approx 1$ is considered. In Eq. 11), as the isotropic and linear elastic material condition is applied, the resultant force, moment, and transverse shear force can be respectively expressed as:

12 $$\begin{array}{rcl}{n}^{\alpha \beta } & = & {\int }_{-h/2}^{h/2}{S}^{\alpha \beta }d{\theta }^{3}={B}^{\alpha \beta \gamma \mu }h{\varepsilon }_{\gamma \mu }\\ {m}^{\alpha \beta } & = & {\int }_{-h/2}^{h/2}{S}^{\alpha \beta }{\theta }^{3}d{\theta }^{3}={B}^{a\beta \gamma \mu }\frac{{h}^{3}}{12}{\kappa }_{\gamma \mu }\\ {q}^{\alpha } & = & {\int }_{-h/2}^{h/2}{S}^{\alpha 3}d{\theta }^{3}=k{B}^{\alpha 3\beta 3}h{\gamma }_{\beta }\\ {B}^{\alpha \beta \gamma \mu } & = & \frac{E}{2(1+\nu )}({a}^{\alpha \gamma }{a}^{\beta \mu }++{a}^{\alpha \mu }{a}^{\beta \gamma }+\frac{2\nu }{1-\nu }{a}^{\alpha \beta }{a}^{\gamma \mu })\\ {B}^{\alpha 3\beta 3} & = & \frac{E}{2(1+\nu )}{a}^{\alpha \beta }\end{array}$$

where, *k* is the transverse shear correction factor that is determined to be 5/6.

By applying the virtual work principle, the variational term of the internal energy can be obtained as:

13 $$\delta U={\int }_{A}{n}^{\alpha \beta }\delta {\varepsilon }_{\alpha \beta }+{m}^{\alpha \beta }\delta {\kappa }_{\alpha \beta }+{q}^{\alpha }\delta {\gamma }_{\alpha }\sqrt{a}dA.$$

The strain measure variational terms are obtained from the directional derivative formulation of virtual displacement and virtual rotations. Then, they are calculated as:

14 $$\begin{array}{rcl}\delta {\varepsilon }_{\alpha \beta } & = & \frac{1}{2}({\overrightarrow{x}}_{,\alpha }\cdot \delta {\overrightarrow{u}}_{,\beta }+{\overrightarrow{x}}_{,\beta }\cdot \delta {\overrightarrow{u}}_{,\alpha })\\ \delta {\kappa }_{\alpha \beta } & = & \frac{1}{2}({\overrightarrow{x}}_{,\alpha }\cdot \delta {\overrightarrow{t}}_{,\beta }+{\overrightarrow{x}}_{,\beta }\cdot \delta {\overrightarrow{t}}_{,\alpha }+\delta {\overrightarrow{u}}_{,\alpha }\cdot {\overrightarrow{t}}_{,\beta }+\delta {\overrightarrow{u}}_{,\beta }\cdot {\overrightarrow{t}}_{,\alpha })\\ \delta {\gamma }_{\alpha } & = & ({\overrightarrow{x}}_{,\alpha }\cdot \delta \overrightarrow{t}+\delta {\overrightarrow{u}}_{,\alpha }\cdot \overrightarrow{t})\end{array}.$$

The folding deformation of the PS sheet shows large displacement and rotation angle, so it requires non-linear deformation analysis. The iterative Newton-Raphson method is employed for the non-linear problem in this paper, and the tangential stiffness matrix and the internal force vector should be constructed from the variational form of the internal energy. The differentiation for the kinematic variables *x* and *t* is performed, and it follows that:

15 $${\rm{\Delta }}\overrightarrow{x}={\rm{\Delta }}(\overrightarrow{X}+\overrightarrow{u})={\rm{\Delta }}\overrightarrow{u}{\rm{\Delta }}\overrightarrow{t}={\rm{\Delta }}\overrightarrow{t}(\alpha ,\beta )={\rm{\Lambda }}\{\begin{array}{c}{\rm{\Delta }}\alpha \\ {\rm{\Delta }}\beta \end{array}\},{\rm{\Delta }}\overrightarrow{r}={\rm{\Delta }}\overrightarrow{r}(\overrightarrow{x},\overrightarrow{t})={\rm{\Delta }}\overrightarrow{r}(\overrightarrow{u},\overrightarrow{t})$$

where, the matrix ${\rm{\Lambda }}$ is defined as:

16 $${\rm{\Lambda }}=[\begin{array}{cc}\sin (\alpha )\sin (\beta ) & -\cos (\alpha )\cos (\beta )\\ -\cos (\alpha ) & 0\\ -\sin (\alpha )\cos (\beta ) & -\cos (\alpha )\sin (\beta )\end{array}].$$

The tangential stiffness matrix is obtained from the differentiation of the variational internal energy form. The tangential stiffness matrix is usually separated into the geometric part and material part, as:

17 $$\begin{array}{rcl}D\delta U[{\rm{\Delta }}\overrightarrow{r}] & = & {D}_{g}\delta U[{\rm{\Delta }}\overrightarrow{r}]+{D}_{m}\delta U[{\rm{\Delta }}\overrightarrow{r}]\\ {D}_{g}\delta U[{\rm{\Delta }}\overrightarrow{r}] & = & {\int }_{{\rm{\Omega }}}{n}^{\alpha \beta }D\delta {\varepsilon }_{\alpha \beta }[{\rm{\Delta }}\overrightarrow{r}]+{m}^{\alpha \beta }D\delta {\kappa }_{\alpha \beta }[{\rm{\Delta }}\overrightarrow{r}]+{q}^{\alpha }D\delta {\gamma }_{\alpha }[{\rm{\Delta }}\overrightarrow{r}]d{\rm{\Omega }}\\ {D}_{m}\delta U[{\rm{\Delta }}\overrightarrow{r}] & = & {\int }_{{\rm{\Omega }}}D{n}^{\alpha \beta }[{\rm{\Delta }}\overrightarrow{r}]\delta {\varepsilon }_{\alpha \beta }+D{m}^{\alpha \beta }[{\rm{\Delta }}\overrightarrow{r}]\delta {\kappa }_{\alpha \beta }+D{q}^{\alpha }[{\rm{\Delta }}\overrightarrow{r}]\delta {\gamma }_{\alpha }d{\rm{\Omega }}\end{array}$$

The individual derivative terms of the membrane, bending, and transverse shear strain measures in the geometric part are expressed as:

18 $$\begin{array}{rcl}D\delta {\varepsilon }_{\alpha \beta }[{\rm{\Delta }}\overrightarrow{r}] & = & \frac{1}{2}(\delta {\overrightarrow{u}}_{,\alpha }\cdot {\rm{\Delta }}({\overrightarrow{u}}_{,\beta })+{\rm{\Delta }}({\overrightarrow{u}}_{,\alpha })\cdot \delta {\overrightarrow{u}}_{,\beta })\\ D\delta {\kappa }_{\alpha \beta }[{\rm{\Delta }}\overrightarrow{r}] & = & \frac{1}{2}({\rm{\Delta }}({\overrightarrow{u}}_{,\alpha })\cdot \delta {\overrightarrow{t}}_{,\beta }+{\rm{\Delta }}({\overrightarrow{u}}_{,\beta })\cdot \delta {\overrightarrow{t}}_{,\alpha }+\delta {\overrightarrow{u}}_{,\alpha }\cdot {\rm{\Delta }}({\overrightarrow{t}}_{,\beta }))\\  &  & +\delta {\overrightarrow{u}}_{,\beta }\cdot {\rm{\Delta }}({\overrightarrow{t}}_{,\alpha })+{\overrightarrow{x}}_{,\alpha }\cdot D(\delta {\overrightarrow{t}}_{,\beta })[{\rm{\Delta }}\overrightarrow{t}]+{\overrightarrow{x}}_{,\beta }\cdot D(\delta {\overrightarrow{t}}_{,\alpha })[{\rm{\Delta }}\overrightarrow{t}])\\ D\delta {\gamma }_{\alpha }[{\rm{\Delta }}\overrightarrow{r}] & = & ({\rm{\Delta }}({\overrightarrow{u}}_{,\alpha })\cdot \delta \overrightarrow{t}+\delta {\overrightarrow{u}}_{,\alpha }\cdot {\rm{\Delta }}\overrightarrow{t}+{\overrightarrow{x}}_{,\alpha }\cdot D(\delta \overrightarrow{t})[{\rm{\Delta }}\overrightarrow{t}])\end{array}.$$

When the finite element discretization is applied to the geometric tangential stiffness matrix, the terms are formulated as:

19 $$\begin{array}{rcl}{n}^{\alpha \beta }D\delta {\varepsilon }_{\alpha \beta }[{\rm{\Delta }}\overrightarrow{r}] & = & \sum _{i=1}^{4}\sum _{j=1}^{4}\delta {\overrightarrow{u}}_{i}\cdot {\rm{\Delta }}{\overrightarrow{u}}_{j}\frac{1}{2}{n}^{\alpha \beta }({N}_{,\alpha }^{i}{N}_{,\beta }^{j}+{N}_{,\alpha }^{j}{N}_{,\beta }^{i})\\ {m}^{\alpha \beta }D\delta {\kappa }_{\alpha \beta }[{\rm{\Delta }}\overrightarrow{r}] & = & \sum _{i=1}^{4}\sum _{j=1}^{4}\delta {\overrightarrow{t}}_{i}\cdot {\rm{\Delta }}{\overrightarrow{u}}_{j}\frac{1}{2}{m}^{\alpha \beta }({N}_{,\alpha }^{i}{N}_{,\beta }^{j}+{N}_{,\alpha }^{j}{N}_{,\beta }^{i})\\  &  & +\sum _{i=1}^{4}\sum _{j=1}^{4}\delta {\overrightarrow{u}}_{i}\cdot {\rm{\Delta }}{\overrightarrow{t}}_{j}\frac{1}{2}{m}^{\alpha \beta }({N}_{,\alpha }^{i}{N}_{,\beta }^{j}+{N}_{,\alpha }^{j}{N}_{,\beta }^{i})\\  &  & +\sum _{i=1}^{4}{\rm{\Delta }}(\delta {\overrightarrow{t}}_{i})\cdot \frac{1}{2}{m}^{\alpha \beta }({\overrightarrow{x}}_{,\alpha }{N}_{,\beta }^{i}+{\overrightarrow{x}}_{,\beta }{N}_{,\alpha }^{i})\\ {q}^{\alpha }D\delta {\gamma }_{\alpha }[{\rm{\Delta }}\overrightarrow{r}] & = & \sum _{i=1}^{4}\sum _{j=1}^{4}\delta {\overrightarrow{t}}_{i}\cdot {\rm{\Delta }}{\overrightarrow{u}}_{j}({q}^{\alpha }{N}^{i}{N}_{,\alpha }^{j})\\  &  & +\sum _{i=1}^{4}\sum _{j=1}^{4}\delta {\overrightarrow{u}}_{i}\cdot {\rm{\Delta }}{\overrightarrow{t}}_{j}({q}^{\alpha }{N}_{,\alpha }^{i}{N}^{j})\\  &  & +\sum _{i=1}^{4}{\rm{\Delta }}(\delta {\overrightarrow{t}}_{i})\cdot ({q}^{\alpha }{\overrightarrow{x}}_{,\alpha }{N}^{i})\end{array}.$$

In addition, the strain measure derivatives of the geometric tangential stiffness matrix in Eq. 17) are obtained as:

20 $$\begin{array}{rcl}D{n}^{\alpha \beta }[{\rm{\Delta }}\overrightarrow{r}]\delta {\varepsilon }_{\alpha \beta } & = & {B}^{\alpha \beta \gamma \mu }h{\rm{\Delta }}{\varepsilon }_{\gamma \mu }\delta {\varepsilon }_{a\beta }\\ D{m}^{\alpha \beta }[{\rm{\Delta }}\overrightarrow{r}]\delta {\kappa }_{\alpha \beta } & = & {B}^{a\beta \gamma \mu }\frac{{h}^{3}}{12}{\rm{\Delta }}{\kappa }_{\gamma \mu }\delta {\kappa }_{\alpha \beta }\\ D{q}^{\alpha }[{\rm{\Delta }}\overrightarrow{r}]\delta {\gamma }_{\alpha } & = & k{B}^{\alpha 3\beta 3}h{\rm{\Delta }}{\gamma }_{\beta }\delta {\gamma }_{\alpha }\end{array},$$

where, ${B}^{ijkl}$ is the 4th order constitutive tensor expressed in Eq. 12). ${B}^{ijkl}$ is determined by the constitutive relationship between the strain and stress, so it is changed via the stress that the material is given. However, when the polystyrene sheet is folded by light-absorption, the major deformation occurs at the black-coloured line pattern. The non-printed area of the polystyrene sheet, where the shell element is applied, does not generate large strain. Therefore, the linear elastic model is assumed in the non-printed area of the polystyrene sheet.

### Cohesive line element

The folding deformation has been expressed by using the bending deformation considering the cohesive line element, damage model, crack and hinge line, due to its localized deformation and discontinuous rotation^38,39,52–55^. In the folding deformation analysis, this section employs the cohesive line element, among the various analysis methods for folding deformation. The cohesive line element provides additional nodes at the folded line, and then the discontinued rotation angles generated at the folded line can be handled without difficulty. This section introduces the cohesive line element used in Giampieri *et al*.’s study^38,39^.

**Figure 11:**
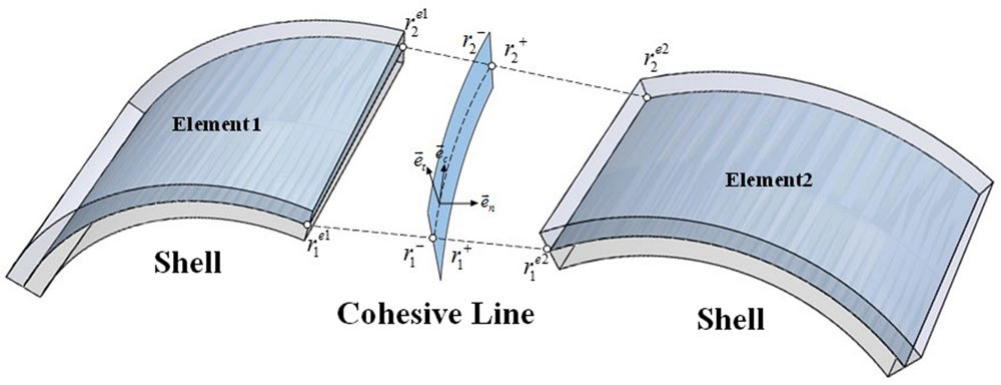
Shell element and cohesive line element.

In the cohesive line element, the additional nodes are defined at the previous existing nodes. Figure 11 shows that accordingly, the nodes have the same coordinates.

The coordinate system ${\overrightarrow{e}}_{i}$ in the cohesive line element is the co-rotation frame defined in the reference interface surface. In addition, the kinematic variables at the line are interpolated by the linear shape function $N(\zeta )$; the position vector at the line is then expressed as:

21 $${\overrightarrow{R}}_{L}={\overrightarrow{X}}_{L}(\zeta )+{\theta }^{3}{\overrightarrow{T}}_{L}(\zeta ).$$

Also, the covariant base vector in the undeformed configuration can be defined as:

22 $$\begin{array}{rcl}{\overrightarrow{G}}_{c} & = & {\overrightarrow{X}}_{{\rm{L}},{\rm{c}}}+{\theta }^{3}{\overrightarrow{T}}_{L,c}\\ {\overrightarrow{G}}_{3} & = & {\overrightarrow{T}}_{L}\\ {\overrightarrow{G}}_{n} & = & \frac{{\overrightarrow{G}}_{c}\times {\overrightarrow{G}}_{3}}{|{\overrightarrow{G}}_{c}\times {\overrightarrow{G}}_{3}|}\end{array},$$

where, ${\overrightarrow{G}}_{n}$ is obtained from the other two orthogonal covariant vectors.

The cohesive line element allows the displacement jump for the discontinuous rotation angles. The displacement jump at the interface line is expressed as:

23 $$[\kern-2pt[ \overrightarrow{w}]\kern-2pt] ={\overrightarrow{w}}^{+}-{\overrightarrow{w}}^{-}=[\kern-2pt[ \overrightarrow{u}]\kern-2pt] +{\theta }^{3}[\kern-2pt[ \overrightarrow{t}]\kern-2pt] .$$

Also, the deformed position vector and shell director at the line are defined as:

24 $$\overrightarrow{\tilde{r}}=\frac{1}{2}({\overrightarrow{r}}^{+}+{\overrightarrow{r}}^{-})=\overrightarrow{\tilde{x}}+{\theta }^{3}\overrightarrow{\tilde{t}}\overrightarrow{\tilde{x}}=\frac{1}{2}({\overrightarrow{x}}^{+}+{\overrightarrow{x}}^{-}),\overrightarrow{\tilde{t}}=\frac{1}{2}({\overrightarrow{t}}^{+}+{\overrightarrow{t}}^{-}).$$

From Eq. 24, the covariant basis vector for the deformed interface surface is expressed as:

25 $$\begin{array}{rcl}{\overrightarrow{\tilde{g}}}_{c} & = & {\overrightarrow{\tilde{x}}}_{,c}+{\theta }^{3}{\overrightarrow{\tilde{t}}}_{,c}.\\ {\overrightarrow{\tilde{g}}}_{3} & = & \overrightarrow{\tilde{t}}\\ {\overrightarrow{\tilde{g}}}_{n} & = & \frac{{\overrightarrow{\tilde{g}}}_{c}\times {\overrightarrow{\tilde{g}}}_{3}}{|{\overrightarrow{\tilde{g}}}_{c}\times {\overrightarrow{\tilde{g}}}_{3}|}\end{array}$$

The total internal energy is separated into the shell element part and the cohesive line element part, and it is shown as:

26 $$\begin{array}{rcl}U & = & {U}_{Shell}+{U}_{Line}\\ {U}_{Line} & = & {\int }_{-\frac{h}{2}}^{\frac{h}{2}}{\int }_{{A}_{L}}\frac{1}{2}\overrightarrow{P}\,\cdot \,\overrightarrow{w}d{A}_{L}d{\theta }^{3},\\ \delta {U}_{Line} & = & {\int }_{-\frac{h}{2}}^{\frac{h}{2}}{\int }_{{A}_{L}}\overrightarrow{P}\cdot \delta \overrightarrow{w}d{A}_{L}d{\theta }^{3}\end{array}$$

where, $\overrightarrow{P}$ and $\overrightarrow{w}$ are the internal stress and strain, respectively, at the cohesive line. The variational form of internal energy can be easily formulated. The variational term of the displacement jump is expressed as:

27 $$\delta [\kern-2pt[ \overrightarrow{w}]\kern-2pt] =\delta [\kern-2pt[ \overrightarrow{u}]\kern-2pt] +{\theta }^{3}\delta [\kern-2pt[ \overrightarrow{t}]\kern-2pt] ,$$

where, the virtual shell director jump is:

28 $$\delta [\kern-2pt[ \overrightarrow{t}]\kern-2pt] =\delta {\overrightarrow{t}}^{+}-\delta {\overrightarrow{t}}^{-}=\delta {\overrightarrow{\vartheta }}^{+}\times {\overrightarrow{t}}^{+}-\delta {\overrightarrow{\vartheta }}^{-}\times {\overrightarrow{t}}^{-}.$$

In Eq. 28, $\overrightarrow{\vartheta }$ denotes the rotation axis vector, which is expressed as:

29 $$\begin{array}{rcl}\delta \overrightarrow{T} & = & {\rm{\Omega }}(\delta \overrightarrow{{\rm{\Theta }}})T=\delta \overrightarrow{{\rm{\Theta }}}\times \overrightarrow{T}\\ \delta \overrightarrow{t} & = & {\rm{\Omega }}(\delta \overrightarrow{\vartheta })t=\delta \overrightarrow{\vartheta }\times t\end{array},$$

where, ${\rm{\Omega }}$ denotes the skew-symmetric tensor.

If $\overrightarrow{\tilde{\vartheta }}=\frac{1}{2}({\overrightarrow{\vartheta }}^{+}+{\overrightarrow{\vartheta }}^{-})$ is defined, $\delta [\kern-2pt[ \overrightarrow{w}]\kern-2pt] \,\,{\rm{and}}\,\delta [\kern-2pt[ \overrightarrow{t}]\kern-2pt]$ are induced from Eqs 28 and 29:

30 $$\begin{array}{rcl}\delta [\kern-2pt[ \overrightarrow{w}]\kern-2pt]  & = & \delta [\kern-2pt[ \overrightarrow{u}]\kern-2pt] +{\theta }^{3}\delta [\kern-2pt[ \overrightarrow{\vartheta }]\kern-2pt] \times \tilde{t}+{\theta }^{3}\delta \tilde{\vartheta }\times [\kern-2pt[ \overrightarrow{t}]\kern-2pt] \\ \delta [\kern-2pt[ \overrightarrow{t}]\kern-2pt]  & = & \delta [\kern-2pt[ \overrightarrow{\vartheta }]\kern-2pt] \times \overrightarrow{\tilde{t}}+\delta \overrightarrow{\tilde{\vartheta }}\times [\kern-2pt[ \overrightarrow{t}]\kern-2pt] \end{array}.$$

Then, the variational displacement in the interface line is derived

31 $$\begin{array}{rcl}\delta \overrightarrow{w} & = & \delta [\kern-2pt[ \overrightarrow{u}]\kern-2pt] +{\theta }^{3}\delta [\kern-2pt[ \overrightarrow{\vartheta }]\kern-2pt] \times \overrightarrow{\tilde{t}}-\delta \overrightarrow{\tilde{{\vartheta }}}\times \overrightarrow{u}\\  & = & \delta \overrightarrow{\phi }+{\theta }^{3}\delta [\kern-2pt[ \overrightarrow{\vartheta }]\kern-2pt] \times \overrightarrow{\tilde{t}}\\ \delta \overrightarrow{\phi } & = & \delta \overrightarrow{u}-\delta \overrightarrow{\tilde{\vartheta }}\times \overrightarrow{u}\end{array}.$$

Substituting Eq. 31 into Eq. 26, the variation form of the internal energy $\delta {U}_{Line}$ is separated into the in-plane part and out-of-plane part, as:

32 $$\begin{array}{rcl}\delta {U}_{Line} & = & {\int }_{-\frac{h}{2}}^{\frac{h}{2}}{\int }_{{A}_{L}}\overrightarrow{P}\cdot \delta \overrightarrow{w}d{A}_{L}d{\theta }^{3}\\  & = & {\int }_{{A}_{L}}({\int }_{-\frac{h}{2}}^{\frac{h}{2}}\overrightarrow{P}d{\theta }^{3})\cdot \delta \overrightarrow{\phi }d{A}_{L}\\  &  & +{\int }_{{A}_{L}}({\int }_{-\frac{h}{2}}^{\frac{h}{2}}(\overrightarrow{\tilde{t}}\times \overrightarrow{P}){\theta }^{3}d{\theta }^{3})\cdot \delta [\kern-2pt[ \overrightarrow{\vartheta }]\kern-2pt] d{A}_{L}\\  & = & {\int }_{{A}_{L}}\overrightarrow{N}\cdot \delta \overrightarrow{\phi }d{A}_{L}+{\int }_{{A}_{L}}\overrightarrow{M}\cdot \delta [\kern-2pt[ \overrightarrow{\vartheta }]\kern-2pt] d{A}_{L}\end{array},$$

where, $\overrightarrow{N}\,\,{\rm{and}}\,\,\overrightarrow{M}$ are the internal force and moment, respectively, per unit length. The components of $\overrightarrow{N}\,\,{\rm{and}}\,\,\overrightarrow{M}$ are defined as:

33 $$\begin{array}{c}\,{N}_{c}=\overrightarrow{N}\cdot {\overrightarrow{e}}_{c},\,\,\,\,{N}_{n}=\overrightarrow{N}\cdot {\overrightarrow{e}}_{n},\,\,\,\,{N}_{t}=\overrightarrow{N}\cdot {\overrightarrow{e}}_{t}\\ {M}_{c}=\overrightarrow{M}\cdot {\overrightarrow{e}}_{c},\,\,\,{M}_{n}=\overrightarrow{M}\cdot {\overrightarrow{e}}_{n},\,\,\,{M}_{t}=\overrightarrow{M}\cdot {\overrightarrow{e}}_{t}\simeq \overrightarrow{M}\cdot \tilde{t}=0,\end{array}$$

where the drilling moment ${M}_{t}$ is assumed to be zero.

As the shell element, the linearization process of internal energy is performed in the cohesive line element for the non-linear deformation problem. The tangential stiffness is directly obtained from the derivative of the virtual internal energy, and it is separated into geometric part and material part, as:

34 $$\begin{array}{rcl}D\delta {U}_{Line}[{\rm{\Delta }}\overrightarrow{r}] & = & {D}_{g}\delta {U}_{Line}[{\rm{\Delta }}\overrightarrow{r}]+{D}_{m}\delta {U}_{Line}[{\rm{\Delta }}\overrightarrow{r}]\\ {D}_{g}\delta {U}_{Line}[{\rm{\Delta }}\overrightarrow{r}] & = & {\int }_{{A}_{L}}\overrightarrow{N}\cdot D\delta \overrightarrow{\phi }[{\rm{\Delta }}\overrightarrow{r}]d{A}_{L}+{\int }_{{A}_{L}}\overrightarrow{M}\cdot D\delta [\kern-2pt[ \overrightarrow{\vartheta }]\kern-2pt] [{\rm{\Delta }}\overrightarrow{r}]d{A}_{L}\\ {D}_{m}\delta {U}_{Line}[{\rm{\Delta }}\overrightarrow{r}] & = & {\int }_{{A}_{L}}D\overrightarrow{N}[{\rm{\Delta }}\overrightarrow{r}]\cdot \delta \overrightarrow{\phi }d{A}_{L}+{\int }_{{A}_{L}}D\overrightarrow{M}[{\rm{\Delta }}\overrightarrow{r}]\cdot \delta [\kern-2pt[ \overrightarrow{\vartheta }]\kern-2pt] d{A}_{L}.\end{array}.$$

Because $\delta \overrightarrow{u}$ and $\delta \overrightarrow{\vartheta }$ are the primary kinematic variables, $D\delta \overrightarrow{u}=D\delta [\kern-2pt[ \overrightarrow{\vartheta }]\kern-2pt] =D\delta \overrightarrow{\tilde{\vartheta }}=0$ can be established. Then, the geometric part in Eq. 34 is written as:

35 $${D}_{g}\delta {U}_{Line}[{\rm{\Delta }}\overrightarrow{r}]=-{\int }_{{A}_{L}}N\cdot (\delta \overrightarrow{\tilde{\vartheta }}\times {\rm{\Delta }}\overrightarrow{u})d{A}_{L},$$

and each term of the material tangential stiffness matrix is formulated as:

36 $${D}_{m}\delta {U}_{Line}[{\rm{\Delta }}\overrightarrow{r}]={\int }_{{A}_{L}}{\rm{\Delta }}\overrightarrow{N}\cdot \delta \overrightarrow{\phi }d{A}_{L}+{\int }_{{A}_{L}}{\rm{\Delta }}\overrightarrow{M}\cdot \delta [\kern-2pt[ \overrightarrow{\vartheta }]\kern-2pt] d{A}_{L},$$

where, $${\rm{\Delta }}N\,{\rm{and}}\,{\rm{\Delta }}M$$ are:

37 $$\begin{array}{rcl}{\rm{\Delta }}\overrightarrow{N} & = & {\int }_{-\frac{h}{2}}^{\frac{h}{2}}{\rm{\Delta }}\overrightarrow{P}d{\theta }^{3}\\ {\rm{\Delta }}\overrightarrow{M} & = & {\int }_{-\frac{h}{2}}^{\frac{h}{2}}({\rm{\Delta }}\overrightarrow{\tilde{t}}\times \overrightarrow{P}+\overrightarrow{\tilde{t}}\times {\rm{\Delta }}\overrightarrow{P}){\theta }^{3}d{\theta }^{3}\end{array}.$$

In the light-activated folding behaviour of polystyrene sheet, the main deformations are the vertical deformation, and the rotation angle on the cohesive line axis. Accordingly, the tangential moduli matrix of the cohesive line element is given as:

38 $${C}_{Line}=[\begin{array}{cccccc}{c}_{n} & 0 & 0 & 0 & {c}_{n{\vartheta }_{c}} & 0\\  & {c}_{c} & 0 & 0 & 0 & 0\\  &  & {c}_{t} & 0 & 0 & 0\\  &  &  & {c}_{{\vartheta }_{n}} & 0 & 0\\ sym &  &  &  & {c}_{{\vartheta }_{c}} & 0\\  &  &  &  &  & 0\end{array}],$$

where, ${c}_{c},{c}_{t},{c}_{{\vartheta }_{n}},{c}_{n},{c}_{n{\vartheta }_{c}}\,{\rm{and}}\,{c}_{{\vartheta }_{c}}$ are the elastic tangent stiffness in the folded line.

From Eqs 32, 34–36, and 38, the geometric and material terms of the tangential stiffness matrix form are given as:

39 $$\begin{array}{rcl}{D}_{g}\delta {U}_{Line}[{\rm{\Delta }}\overrightarrow{r}] & = & {\int }_{{\Omega }_{L}}\delta {[\kern-2pt[ \overrightarrow{r}]\kern-2pt] }^{T}\tilde{B}[{\rm{\Omega }}(\overrightarrow{N})]\bar{B}{\rm{\Delta }}[\kern-2pt[ \overrightarrow{r}]\kern-2pt] d{{\rm{\Omega }}}_{L}\\ {D}_{m}\delta {U}_{Line}[{\rm{\Delta }}\overrightarrow{r}] & = & {\int }_{{\Omega }_{L}}\delta {[\kern-2pt[ \overrightarrow{r}]\kern-2pt] }^{T}{[\begin{array}{c}{B}_{m}\\ {B}_{b}\end{array}]}^{T}{C}_{Line}[\begin{array}{c}{B}_{m}\\ {B}_{b}\end{array}]{\rm{\Delta }}[\kern-2pt[ \overrightarrow{r}]\kern-2pt] d{{\rm{\Omega }}}_{L}\end{array}.$$

In Eq. 39, $\tilde{B},\bar{B},{B}_{m}\,{\rm{and}}\,{B}_{b}$ have relationships as:

40 $$[\tilde{B}]=\frac{1}{2}[\begin{array}{cccc}0 & 0 & \{{\overrightarrow{e}}_{n}\cdot {\rm{\Omega }}({\overrightarrow{t}}^{+})\} & \{{\overrightarrow{e}}_{n}\cdot {\rm{\Omega }}({\overrightarrow{t}}^{-})\}\\ 0 & 0 & \{{\overrightarrow{e}}_{c}\cdot {\rm{\Omega }}({\overrightarrow{t}}^{+})\} & \{{\overrightarrow{e}}_{c}\cdot {\rm{\Omega }}({\overrightarrow{t}}^{-})\}\\ 0 & 0 & \{{\overrightarrow{e}}_{t}\cdot {\rm{\Omega }}({\overrightarrow{t}}^{+})\} & \{{\overrightarrow{e}}_{t}\cdot {\rm{\Omega }}({\overrightarrow{t}}^{-})\}\end{array}],$$

41 $$[\bar{B}]=[\begin{array}{cccc}\{{\overrightarrow{e}}_{n}\} & -\{{\overrightarrow{e}}_{n}\} & 0 & 0\\ \{{\overrightarrow{e}}_{c}\} & -\{{\overrightarrow{e}}_{c}\} & 0 & 0\\ \{{\overrightarrow{e}}_{t}\} & -\{{\overrightarrow{e}}_{t}\} & 0 & 0\end{array}],$$

42 $$[{B}_{m}]=[\begin{array}{cccc}\{{\overrightarrow{e}}_{n}\} & -\{{\overrightarrow{e}}_{n}\} & \frac{1}{2}\{{\overrightarrow{e}}_{n}\cdot {\rm{\Omega }}(u){\rm{\Omega }}({t}^{+})\} & \frac{1}{2}\{{\overrightarrow{e}}_{n}\cdot {\rm{\Omega }}(u){\rm{\Omega }}({t}^{-})\}\\ \{{\overrightarrow{e}}_{c}\} & -\{{\overrightarrow{e}}_{c}\} & \frac{1}{2}\{{\overrightarrow{e}}_{c}\cdot {\rm{\Omega }}(u){\rm{\Omega }}({t}^{+})\} & \frac{1}{2}\{{\overrightarrow{e}}_{c}\cdot {\rm{\Omega }}(u){\rm{\Omega }}({t}^{-})\}\\ \{{\overrightarrow{e}}_{t}\} & -\{{\overrightarrow{e}}_{t}\} & \frac{1}{2}\{{\overrightarrow{e}}_{t}\cdot {\rm{\Omega }}(u){\rm{\Omega }}({t}^{+})\} & \frac{1}{2}\{{\overrightarrow{e}}_{t}\cdot {\rm{\Omega }}(u){\rm{\Omega }}({t}^{-})\}\end{array}],$$

43 $$[{B}_{b}]=[\begin{array}{cccc}0 & 0 & \{{\overrightarrow{e}}_{n}\cdot {\rm{\Omega }}({t}^{+})\} & \{{\overrightarrow{e}}_{n}\cdot {\rm{\Omega }}({t}^{-})\}\\ 0 & 0 & \{{\overrightarrow{e}}_{c}\cdot {\rm{\Omega }}({t}^{+})\} & \{{\overrightarrow{e}}_{c}\cdot {\rm{\Omega }}({t}^{-})\}\\ 0 & 0 & 0 & 0\end{array}],$$

where, the displacement-strain relationship operator matrices in Eqs 23–26 are discretized by the interpolation shape function $N(\zeta )$.
